# Une localisation rare du fibrome chondromyxoide: à propos d’un cas

**DOI:** 10.11604/pamj.2017.26.219.12289

**Published:** 2017-04-24

**Authors:** Mustafa Nkaoui, Mustapha Mahfoud

**Affiliations:** 1 Service de Chirurgie Orthopédique et de Traumatologie, CHU Ibn Sina, Université Mohammed V Souissi, Rabat, Maroc

**Keywords:** Chondromyxoide, calcanéum, curetage, greffe, Chondromyxoide, calcaneum, curettage, graft

## Images in medicine

Nous rapportons le cas d'une patiente âgée de 65 ans, qui consulte pour douleurs de la cheville droite évoluant depuis environ 15 ans, sans notion de traumatisme initial. L'examen clinique trouve une cheville droite douloureuse légèrement tuméfiée avec boiterie à la marche nécessitant l'utilisation d'une canne. L'état général est conservé avec un bilan biologique infectieux normal. Le bilan radiologique standard de la cheville droite montre une lésion lytique cloisonnée avec ostéocondensation périphérique du calcanéum, et érosion de la corticale postérieure (A). À l'IRM, la lésion est en hyposignal en T1, Hypersignal hétérogène en T2 (B). Un curetage-comblement par ciment avec greffe autologue prélevée au niveau de la crête iliaque a été réalisé (C, D,E). L'examen anatomopathologique de la pièce a conclu à un fibrome chondromyxoïde du calcanéum. A 6 mois de recul, la patiente est autonome marchant avec légère boiterie sans utilisation de cannes et sans douleur.

**Figure 1:**
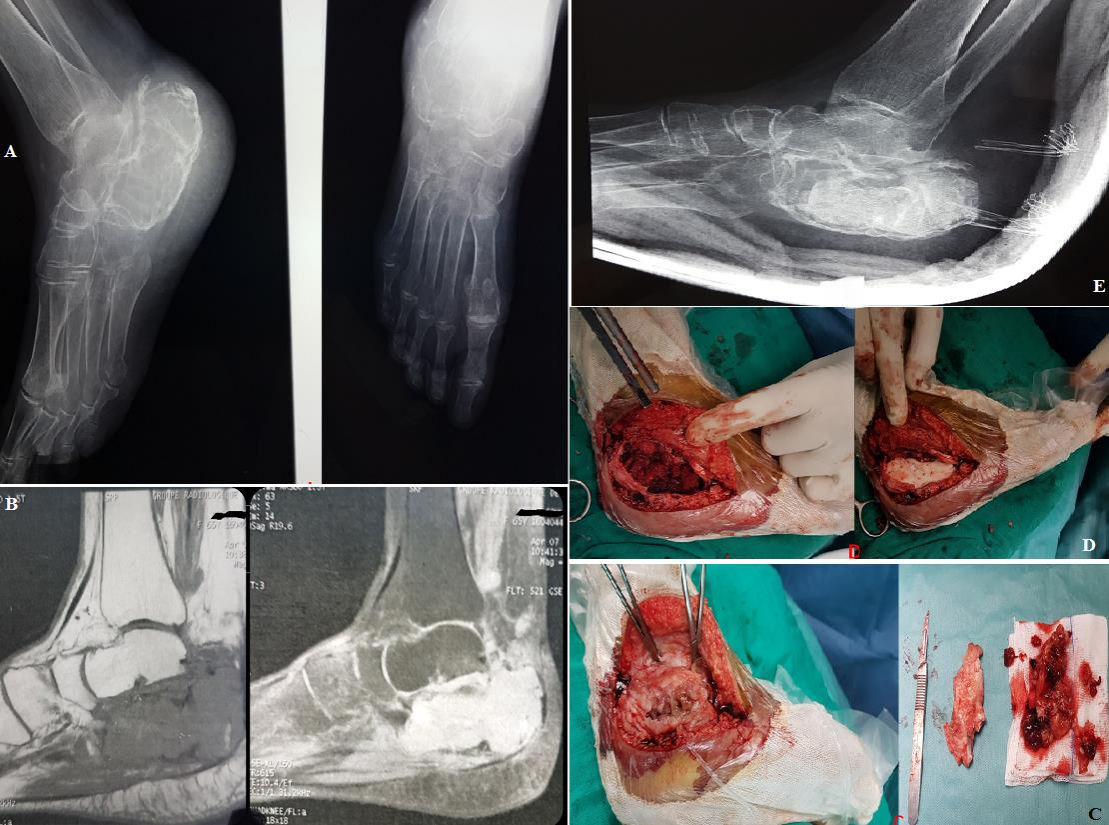
(A) radiographie du pied+cheville droits (face +profil): lyse osseuse géographique du calcanéum, cloisonnée, avec ostéocondensation périphérique et lyse de la corticale postérieure; (B) IRM de la cheville droite montrant une lésion multiloculaire, en hyposignal en T1, hypersignal en T2, avec un contenu hétérogène; (C) image en peropératoire de la lésion; (D) comblement après curetage, par greffe autologue et ciment; (E) radiographie de contrôle de la cheville droite en post opératoire

